# Sibling Jealousy and Temperament: The Mediating Effect of Emotion Regulation in China During COVID-19 Pandemic

**DOI:** 10.3389/fpsyt.2021.729883

**Published:** 2021-10-08

**Authors:** Guoying Qian, Ruonan Li, Wanqi Yang, Ranran Li, Li Tian, Gang Dou

**Affiliations:** ^1^College of Preschool Education, Capital Normal University, Beijing, China; ^2^School of Psychology, Capital Normal University, Beijing, China; ^3^School of Education, Hubei University of Arts and Science, Xiangyang, China

**Keywords:** preschool first-born children, temperament, emotion regulation, sibling jealousy, COVID-19 pandemic

## Abstract

This study aimed to examine first-born children's sibling jealousy and explore the relationships among first-born children's sibling jealousy, temperament, and emotion regulation in China during COVID-19 pandemic. The research hypotheses of this study are empirically examined through online and offline surveys. A sample of 304 two-child families from China participated in the study; the first-born children were aged between 1.17 and 7 years. The results indicated the following: (1) the older the first-born children and the greater the age difference between siblings, the lower the sibling jealousy. (2) Difficult temperament of first-born children could predict sibling jealousy significantly and positively, and emotion regulation could predict sibling jealousy negatively. (3) There was a partially mediating effect of emotion regulation between temperament and sibling jealousy. Compared with intermediate temperament, first-born children with difficult temperament had weaker emotion regulation and higher sibling jealousy. Overall, findings have important implications for psychological interventions for families of first-born children with difficult temperament.

## Introduction

The COVID-19 pandemic has greatly changed people's way of life and had a profound impact on people's psychology and behavior. In order to avoid the further spread of COVID-19, many countries, including China, have taken strict containment measures such as quarantine, closure of schools and public entertainment places, and social distancing (1, 2). During the normalization of the COVID-19 pandemic prevention and control in China, although the children have returned to school, they no longer go to public playgrounds and interest classes after school and during holidays as usual. Thus, they spend more time with their families.

Benefiting from the newly revised family planning policy, many Chinese families are transforming from one-child families into two-child families. And in the coming future, even more and more families with three children will appear. Due to the changes in family structure, sibling competition and sibling jealousy have become high-frequency issues of social concern, as well as practical problems faced by many young parents. The birth of the second child often causes some physical and psychological problems compared to that of the first (3, 4). Compared to the parents of two or more children, parents of only one child need to pay special attention to a new type of relationship that emerges after the birth of the second child, the sibling relationship, which was found in Previous studies can be influenced by the factors including sibling factors themselves (e.g., structural characteristics, temperament) and parent factors (parent-child relations, differential treatment, marital relationship, etc.) (5).

Sibling relationships might influence children's development through the mechanism of attachment, social learning, and social comparison (5). Siblings often develop a range of problematic behaviors due to jealousy (6–8). In the period of the normalization of the COVID-19 pandemic prevention and control, Children of two-child families are spending more time getting along with each other at home than before. Parents not only need to work, but also spend more time taking care of their children. They bear greater pressure and are more prone to anxiety and irritability, resulting in inappropriate treatment of their children (9). According to Volling et al. (8), first-born children may be jealous of their younger siblings because their parents spend more time with or take more care of the latter. Therefore, studying sibling jealousy in Chinese two-child families currently has special significance for the healthy development of children.

From a relationship perspective, jealousy refers to the emotion that an individual experiences when his or her important relationship with someone is threatened or faced with loss due to the intervention of a third party. Jealousy usually exists in the context of a social triangle formed by the jealous, beloved, and rival (10–12). As an emotion with a specific function, the purpose of jealousy is to prevent others from taking away the intimacy formed within relations, and the jealous person needs to take certain actions to protect this relationship (13). In family relationships, sibling jealousy refers to the complex social emotions that arise when the intimate relationship between a child and their parents is threatened by another child (12, 14). To restore intimacy, children may approach and seek the attention of their loved ones, become hostile to their siblings, or even prevent their siblings from having contact with their parents (15). At the psychological level, first-born children usually show emotions such as sadness, anxiety, and anger, and on the behavioral level, there will be manifestations such as seeking closeness, yelling at someone, degradation of behaviors, sleep changes, violent behavior, and decreased appetite (16–18). In families with multiple children, sibling jealousy is more likely to occur because of the relative reduction in the interaction time between the mother and one of the children, the unstable quality of attachment security, and the differentiated treatment of parents (18–20). Transactional models hold that the degree of sibling jealousy is primarily influenced by several factors, including individual characteristics, parental characteristics, family relations, and social environment (21). In this study, we focused on the influence of temperament and emotion regulation on sibling jealousy.

Researchers have found that girls' siblings are more jealous than boys' (22–24), and the age of the first child is negatively correlated with jealousy (25). As far as siblings are concerned, different gender combinations and age differences provide a unique family background for children's social adaptation process in China (5) and are also an important factor for siblings to compete for the limited resources of their parents (26). However, studies have found that the age gap and gender combination are not associated with sibling jealousy (24, 27). Therefore, it is necessary to study the influence of age, gender, gender combination and age gap on the jealousy of first-born children in the Chinese test group.

The temperament of children refers to the individual differences in the emotion, activity level, and attention of children in the early stage, and it is the external manifestation of children's response to the surrounding stimulation. It is more of an innate factor that is less affected by the environment. The temperament of young children seems to be the key factor in understanding the difficulties children face in the transition to sibling relationships and whether sibling jealousy appears in early childhood (28). Bad-tempered children are more likely to protest their mothers taking care of the younger siblings after their birth (29). Children with difficult temperaments tend to be less self-soothing, show more negative emotions such as anger, and are reluctant to take care of younger siblings (30, 31). First-born children with difficult temperaments show clinginess, withdrawal, and problems with eating and sleeping during the transition to sibling relationships (31–35).

Emotion regulation is the internal and external process of regulating, evaluating, and managing emotional responses to achieve expected goals, such as controlling emotional expression and effectively managing negative emotions (36). It is also a process in which individuals exert influence on the occurrence, experience, and expression of emotions (37). Children's emotion regulation includes the process of emotion regulation and emotional instability (38). The effect of emotion regulation on jealousy has been confirmed by many researchers (39–41). For example, researchers have found that the use of diversion strategies can reduce the level of pain after jealousy, which shows that effective emotion regulation strategies can reduce negative emotional experiences associated with jealousy (42). In the “More Fun with Sisters and Brothers (MFWSB)” program, researchers have asked children aged 4-8 to learn strategies (e.g., recognition, monitoring, evaluation, and correction) to improve their emotional and social abilities. The results showed that the emotion regulation ability and sibling relationship of the experimental group improved (43). Some researchers also believe that the first child's emotional regulation ability is higher than that of the second child. At the same time, jealousy is more implicit and flexible (27, 40).

There are some commonalities in the development of children's emotion regulation, but there are still some significant individual differences. One of these inherent differences is temperament, defined by Rothbart and Bates (44). Rothbart and Bates believe that temperament is a biological basis that leads to differences in individual behavior, and it will be affected by heredity, maturity, and experience (44). Murphy's research has found that children with more negative emotions and low control do not show stronger jealousy, which he believes is related to the individual's good emotion regulation ability (45). In early childhood, as a part of the temperament, children's emotions and emotion regulation are still affected by individual inherent genetic factors (46). Therefore, the abnormal development of different levels of emotion regulation displayed by children can be traced back to differences in children's inherent temperament (47). Temperament can predict and hinder the development of young children's emotion regulation (48).

**Figure 1:**
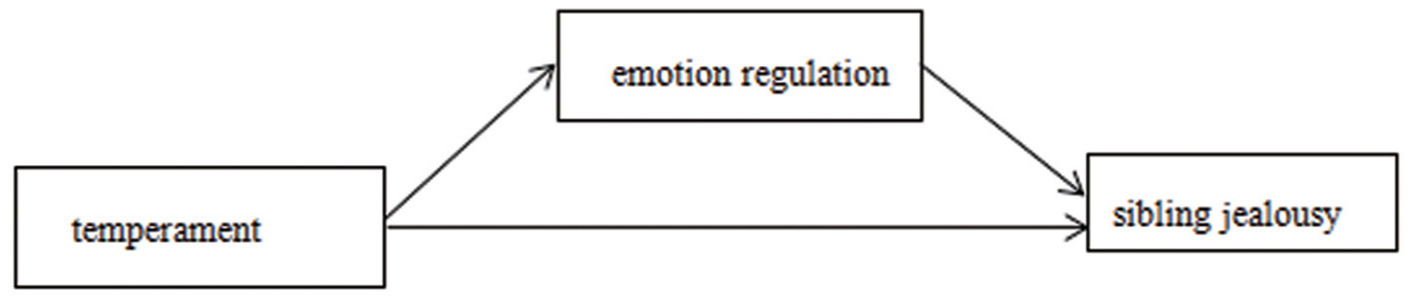
The proposed mediation model.

Based on the above research, we hypothesize that temperament and emotion regulation can jointly predict sibling jealousy and that emotion regulation plays a mediating role in temperament and sibling jealousy (see Figure 1).

## Methods

### Participants

This study was approved by the Research Ethics Committee of the first author's institution. Online and offline surveys were conducted from January to March, 2021. 304 two-child families were randomly recruited from Hubei, Hunan, Jiangxi, and Beijing. The questionnaire was completed by the mothers of the children. There were 149 male and 155 female firstborns, ranging from 1.17 to 7 years of age (*M* = 5.33, *SD* = 1.22), and their younger siblings, including 162 males and 142 females, ranging in from 1.17 to 6.17 years of age (*M* = 2.12, *SD* = 1.32). The average age gap between firstborns and their younger siblings was 3.29 years. Of the families, 70.7% were three-generation families, and 84.5% of the parents were the main caregivers. Families with a combined income of over $10,000 accounted for 86.2% of the total.

### Measures

#### First-Born Children's Sibling Jealousy

A revised Chinese version of the Children *Sibling Jealousy Questionnaire* developed by Kahriman and Kanak (23) and Chen and Yang (49), was used in this study. It consists of 16 items. A five-point scale, ranging from “my child hardly ever does it” to “my child always does it,” was used to estimate the current behaviors and emotions of the first-born child by their main caregiver. The higher the score, the higher the level of sibling jealousy. Cronbach's α for the scale was 0.93.

#### Temperament Scale

The Children's *Temperament Questionnaire* (50, 51) consisted of 72 items belonging to nine subscales: reaction threshold, avoidance, adaptability, activity level, rhythm, reaction intensity, emotional nature, persistence, and the degree of distraction. The scale adopted a seven-point evaluation, ranging from “1” (never) to “7” (always) of the firstborn children's daily behaviors, which were assessed by mothers. Cronbach's α for the scale was 0.94.

#### Emotion Regulation

The *Emotion Regulation Checklist (ERC)* was developed by Shields and Cicchetti (52) and revised by Chang et al. (53) from Hong Kong as well as Chinese mainland scholars (52–54). It consists of 24 items divided into two subscales: (1) emotional instability, which was used to assess children's tendency to be volatile, unstable, and have abnormal negative emotions. The higher the score, the more unstable the mood (52); (2) emotional regulation, which assessed children's ability to respond to emotion regulation in different situations, including the appropriateness of emotional expression, empathy, and emotional self-awareness. The higher the scores, the higher the emotion regulation. The questionnaire was scored on a seven-point scale ranging from “1” (never) to “7” (almost always). Cronbach's α for the scale was 0.92.

### Data Analysis

Data were analyzed using SPSS 22.0. Pearson correlation analysis and the Marco PROCESS (Model 4) (55) were used to analyze the relationships among sibling jealousy, temperament, and emotion regulation.

## Results

### Sibling Jealousy of First-Born Children

A univariate regression analysis was used to investigate the predictability of the age of the first child on the jealousy level toward the siblings. The results showed that *R*^2^= 0.02, indicating that the age of the first child could effectively explain a 2% variation in the jealousy level of the first child toward the sibling. The standardized regression β = −0.15, *t* = −2.61, *p* < 0.05, reaching a significant level, indicated that the greater the age of the first child, the lower the sibling jealousy level, and the unary regression analysis was used to investigate the predictability of the age difference between the first and second children on the sibling jealousy level of the first-born children. In general, *R*^2^= 0.04, which meant that the age difference could effectively explain a 4% variation in the sibling jealousy level of the first child. The standardized regression β = −0.21, *t* = −3.62, *p* < 0.01, reaching a significant level, indicated that the greater the age difference, the lower the sibling jealousy level of the first child.

A univariate analysis of variance (UNIANOVA) with first-born children's sex (two levels) and second-born children's sex (two levels) as factor variables, first-born children's age and the age difference as controlling variables, and the sibling jealousy score as the dependent variable was conducted. There was a significant main effect of the second-born children's sex, *F*_(1, 303)_ = 6.29, *p* < 0.05, $\eta_{p}^{2}$ = 0.02, and the jealousy score of the male second-born children (*M* = 1.92) was significantly higher than that of the female second-born children (M = 1.73). There were no significant main effects of the first-born children's sex [*F*_(1, 303)_ = 0.04, *p* > 0.05, $\eta_{p}^{2}$ = 0.00] and first-born children's sex × second-born children's sex interaction [*F*_(1, 303)_ = 0.10, *p* > 0.05, $\eta_{p}^{2}$ = 0.00] on siblings' jealousy scores.

### Sibling Jealousy, Temperament, and Emotion Regulation

There were four child temperaments: difficult, slow-to-warm, easy, and mixed (51). We set dummy variables for each temperament type, with X1 for “easy to difficult,” X2 for “slow-to-warm-to-difficult,” and X3 for “mixed to difficult.” Pearson correlation analysis was conducted to examine the relationships among sibling jealousy, temperament, and emotion regulation. For the first-born children, sibling jealousy was negatively related to temperament (*r* = −0.19, *p* < 0.01) and emotion regulation (*r* = −0.21, *p* < 0.01), and temperament was positively related to emotion regulation (*r* = 0.34, *p* < 0.01) (see Table 1).

**Table 1:** Pearson correlation coefficients of the study variables (n = 304).

	**M ± SD**	**1**	**2**	**3**
1. Temperament	2.79 ± 1.36	-		
2. Emotion regulation	4.07 ± 0.50	0.34^**^	-	
3. Sibling jealousy	1.83 ± 0.66	−0.19^**^	−0.21^**^	-

** *p < 0.01*.

Based on the correlation analysis results, Model 4 was used to test the mediating effect of emotion regulation on the relationship between temperament and sibling jealousy. Controlling for the gender combination, age difference, family structure, main caregivers, and familial income of the two children, the results (see Table 2) showed that the direct path from X2 and X3 to sibling jealousy (β = −0.24, *p* < 0.05; β = −0.39, *p* < 0.001), in the absence of emotion regulation, was significant. Emotion regulation was significantly associated with X1 (β = 0.56, *p* < 0.001), X2 (β = 0.33, *p* < 0.001), X3 (β = 0.39, *p* < 0.001), and sibling jealousy (β = −0.25, *p* < 0.01). However, only X3 significantly predicted sibling jealousy (β = −0.29, *p* < 0.001). Therefore, compared with intermediate temperament, emotion regulation partially mediated the relationship between difficult temperament and sibling jealousy.

**Table 2:** Testing the mediation effect of emotion regulation on sibling jealousy.

**Predictors (IV)**	**Model 1 (DV: sibling jealousy)**		**Model 2 (DV: emotion regulation)**		**Model 3 (DV: sibling jealousy)**	
	β	*T*	β	*t*	β	*t*
Temperament X1	−0.31	−1.54	0.56	3.77^***^	−0.16	−0.83
Temperament X2	−0.24	−2.02^*^	0.33	3.74^***^	−0.15	−1.31
Temperament X3	−0.39	−4.84^***^	0.39	6.57^***^	−0.29	−3.42^***^
Emotion regulation					−0.25	−3.31^**^
R^2^	0.14 9.55^***^	0.16 11.16^***^	0.17 10.05^***^			
F						

*IV, Independent Variable; DV, Dependent Variable*;

* *p < 0.05*,

** *p < 0.01, and*

*** *p < 0.001*.

To assess the size of the indirect effect and confidence intervals (CIs), a bootstrap procedure was applied. For the indirect effect, 95 percent bias-corrected accelerated CIs without “zero” indicated a significant mediation effect. We generated 5,000 bootstrap samples. The indirect effect of temperament on sibling jealousy mediated by emotion regulation [ab = −0.15, *SE* = 0.06, 95% CI (−0.26, −0.06)] was significant. The mediation effect accounted for 38.68% of the total effect. The 95% CI did not contain zero, showing that temperament exerted a significant indirect effect on sibling jealousy via emotion regulation.

## Discussion

The present research was framed during the normalization of the COVID-19 pandemic, providing unique empirical evidence regarding sibling jealousy and the relationships among first-born children's sibling jealousy, temperament, and emotion regulation. Major results showed that, for first-born children between the ages of 1.17 and 7, the older they are and the greater the age difference between them and the second children, the lower their sibling jealousy. There were no significant main effects for the sex difference in sibling jealousy, which is consistent with previous studies (5, 25, 27). The sibling jealousy of male second-born children was significantly higher than that of females, which was inconsistent with the results of previous studies (22–24). A possible reason was that, in the socio-cultural context of China, older brothers or sisters should give way to younger ones, so the sex difference in the jealousy of the first-born children was not significant, while the male second children had a stronger attachment to their mothers, so their jealousy was higher than that of the females.

Furthermore, this study found that difficult temperament could positively predict sibling jealousy, and emotion regulation was a negative predictor. This result was consistent with previous studies that showed that first-born children with difficult temperaments exhibited more jealous behaviors such as negative emotions, attachment issues, withdrawal, as well as eating and sleeping problems during the transition to sibling relationships (30–35, 56, 57). Children with low emotion regulation who were in a jealous situation would report a higher level of sibling jealousy because of their inability to regulate their own jealousy response (57). Effective emotion regulation could reduce sibling jealousy (27, 42, 43). Although the triadic laboratory paradigm was mainly used to measure sibling jealousy, that is, to design a jealous situation in which a mother or father interacted with one child while ignoring the other, the experimental object was the neglected children, and researchers observed the jealousy emotion and behavior dissonance that were shown in that respective context. In this study, we used a questionnaire to survey the sibling jealousy of first-born children, which involved the first child's anxiety, distress, sadness, anger, bad sleep patterns and eating habits, degradation of behaviors, and attacking the sibling after the birth of the second child. These emotional expressions and behavior performances were consistent with the measurement of sibling jealousy in experimental research. Therefore, the conclusion drawn from the questionnaire survey on Chinese samples was consistent with Western research.

Moreover, this study was the first to explore the mediating role of emotion regulation between temperament and sibling jealousy. As a complex social emotion (12, 14), jealousy is closely related to emotion regulation, while in early childhood, the emotion regulation of children is a part of temperament (46, 47), which, in turn, predicts their emotion regulation (48). Compared with children with intermediate temperament, first-born children with difficult temperament had a weaker emotion regulation ability and higher sibling jealousy. out of 4 domains (i.e., rhythm, avoidance, adaptability, and emotional essence) that were evaluated, difficult children revealed low-scores in at least three domains. They had irregular daily routines, including issues with eating, drinking, sleeping, urinating, and defecating; therefore, children with low temperament rhythms were more likely to be in a changing environment, which was detrimental to their emotional stability. First-born children with an avoidance tendency and poor adaptability to the new environment might be afraid of unfamiliar people and the changes in the environment caused by the new event of the second sibling's birth. Lower scores on emotional nature indicated that first-born children were often in a negative mood, which aggravated the frequency and degree of sibling jealousy occurrence. Therefore, during the normalization of the pandemic, we need to pay special attention to difficult children to help them improve their emotion regulation ability. This will have the effect of reducing sibling jealousy and maintaining a good level of mental health.

This study statically examined the relationships among temperament, emotion regulation, and sibling jealousy during COVID-19 pandemic, but failed to longitudinally investigate the interaction mechanism between siblings in the formation of sibling jealousy and also lacked research on the changes in parenting styles before and after the birth of the second child. Therefore, future research needs to further carry out big data tracking research and in-depth investigations of the interaction mechanism between siblings and that of the parental rearing style and sibling jealousy in different periods. This study used a questionnaire to assess the jealousy toward siblings in first-born children. In the future, it will be necessary to use the triad laboratory paradigm, interviews, and questionnaires to evaluate sibling jealousy more comprehensively and objectively from the perspective of both parents and children.

## Data Availability Statement

The original contributions presented in the study are included in the article/supplementary material, further inquiries can be directed to the corresponding author/s.

## Ethics Statement

The studies involving human participants were reviewed and approved by Scientific Research Ethics Committee of School of preschool education, Capital Normal University. The patients/participants provided their written informed consent to participate in this study.

## Author Contributions

GQ designed the project and supervised the data collection. WY, GQ, and GD collected and analyzed the data. GQ and GD wrote the manuscript with input from all other authors. All authors contributed to the article and approved the submitted version.

## Funding

This study was supported by the Project of Municipal Science and Technology Commission of Beijing (20530290022), Capacity Building for Sci-Tech Innovation-Fundamental Scientific Research Funds (20530290062) and Scientific Research Cultivation Fund of Hubei University of Arts and Science (2017kypy025).

## Conflict of Interest

The authors declare that the research was conducted in the absence of any commercial or financial relationships that could be construed as a potential conflict of interest.

## Publisher's Note

All claims expressed in this article are solely those of the authors and do not necessarily represent those of their affiliated organizations, or those of the publisher, the editors and the reviewers. Any product that may be evaluated in this article, or claim that may be made by its manufacturer, is not guaranteed or endorsed by the publisher.
